# Supplementary material for: Triple-band highly sensitive terahertz metamaterial absorber for biomedical sensing applications
Source: PLoS One. 2025 Aug 19;20(8):e0328077. doi: 10.1371/journal.pone.0328077 (PMC12364324; doi:10.1371/journal.pone.0328077)
Supplement: Attachment — Submitted filename: Response_to_Reviewers.pdf [file pone.0328077.s002.pdf]

# Reviewer

Reviewer #1: This research article presents a metamaterial terahertz absorber for biomedical sensing applications. The paper is well organized but needs a revision before acceptance as follows;

1. The title is suitable.
2. The abstract section presents brief information about the work, it is in a suitable form.
3. The introduction section gives a comprehensive literature review, but it is advised to improve introduction section by following studies:
- https://doi.org/10.1016/j.jmrt.2021.05.031
- https://doi.org/10.1364/OME.447855
- https://doi.org/10.1117/1.OE.63.11.115102
4. Please explain how to obtain geometric design parameters.
5. Please check figures in the main manuscript, it is not seen in the manuscript file directly.
6. Please explain why this operating band was chosen.
7. The conclusion is in a good form.
8. Please mention the experimental application possibilities and future directions.

Reviewer #2: The manuscript presents a triple-band metamaterial absorber (MMA) for terahertz biomedical sensing, achieving high absorption and sensitivity. While the study is comprehensive in scope and simulation-based validation, several limitations and drawbacks should be addressed to enhance the work's scientific rigor and real-world relevance.

1- The entire study is simulation-based, using CST Studio Suite and ADS, without experimental fabrication or measurement.
2- Simulation results, while promising, do not account for real-world fabrication imperfections, material losses, or environmental noise.
3- The study focuses on refractive index sensing of a few biological targets (glucose, malaria, HeLa cells). No analysis is provided on how the sensor performs with mixed samples.
4- As we know, the given absorber is a periodic structure. How to put the test object in?
5- The device operates in 1–6.5 THz, but comparison with existing literature (Table 3, Table 4) often includes devices operating in lower or broader ranges.
6- While some angle-dependent studies are shown, the absorption shifts are noticeable beyond 30°, especially at 60°, where distortions appear.
7- How can we improve the performance of the given sensor.
8- In Fig.1, the basic of information is not clear. The physical model of materials is not given.
9- Please highlight the advance of the study in Introduction. Please explain the development and creative work. The literature review should be carefully considered.
10- Please polish the abstract. Please check the logic of abstract. Please add sentences to explain the meaning, the main points, the improvement and the promising application of the study. Plenty of detail data have given, however, in abstract, important procedures and results should be mentioned in simple manner. Please focus on the main points and the improvement of the study.
11- English in this paper needs improvement, which can make this paper more like a journal paper.

12- Many information without references.
13. The author should elaborate all results with reason.
14. For the explanation of sensors, please add the extra contents or theory to analyze.
15. It is recommended to cite the following articles:
a. https://www.nature.com/articles/s41598-023-46363-x
b. https://iopscience.iop.org/article/10.1088/2040-8986/ac8889/meta
c. https://www.sciencedirect.com/science/article/pii/S2211379720320106
d. https://link.springer.com/chapter/10.1007/978-981-97-0142-1_35

# Reviewer #1:

**Reviewer, Concern #1:** The title is suitable.
**Reviewer, Concern #2:** The abstract section presents brief information about the work, it is in a suitable form.

**Author response:** Thank you very much for your comments on our article. This will motivate us in our future work.

**Reviewer, Concern #3:** The introduction section gives a comprehensive literature review, but it is advised to improve introduction section by following studies:
- https://doi.org/10.1016/j.jmrt.2021.05.031
- https://doi.org/10.1364/OME.447855
- https://doi.org/10.1117/1.OE.63.11.115102

**Author response:** Thank you very much for your comments on our article. Based on your suggestions we have added the suggested literature in the in the introduction section and in the reference section.

**Author action:** We updated the manuscript based on reviewer comments.

For energy harvesting and stealth technology applications, a square-shaped water MTM absorber was suggested [1]. The absorber showed absorption value of more than 90% from frequencies ranges 10.4 to 30 GHz. A triple-band mtm absorber has been proposed for use in biological sensing, filtration, etc. [2]. All of the peaks exhibit good absorption values of 98%, 91%, and 98%, and these peaks also have good sensitivity values. A ultra thin absorber made with combination of SRR is demonstrated [3] for imaging and stealth applications. The size of this structure is very thin and found six resonance peaks.

**Reviewer, Concern #4:** Please explain how to obtain geometric design parameters.

**Author response:** Thank you very much for your comments on our article. Based on your suggestion, we have revised the first paragraph of the ***Materials and Methods*** section to provide a clear understanding of the geometric design parameters.

**Author action:** We updated the manuscript based on reviewer comments.

The height of the front part ,which is also made with gold, is 1 um. The detailed parameter values for all the structures are shown in Table 1. Through an analysis of prior research on comparable metamaterial absorber designs, initial values for characteristics such as substrate width, height, octagonal width, and gap were determined. In addition, a thorough parametric analysis was carried out to optimise important parameters as needed to achieve high absorption efficiency and improved sensing performance.

**Reviewer, Concern #5:** While Please check figures in the main manuscript, it is not seen in the manuscript file directly.

**Author response:** Thank you for pointing this out. We sincerely apologize for the inconvenience. Due to the journal's submission guidelines, all figures were required to be uploaded separately. As a result, the figures have been automatically appended to the end of the PDF file by the submission system. We confirm that all figures referenced in the manuscript are included at the end of the file for review.

**Reviewer, Concern #6:** Please explain why this operating band was chosen.

**Author response:** Thank you very much for your comments on our article. Based on your suggestion, we have made revisions to the second paragraph of the ***Materials and Methods*** section to provide an idea about the operating band.

**Author action:** We updated the manuscript based on reviewer comments.

Terahertz (THz) waves, spanning 0.1–10 THz, combine properties of both millimeter and infrared waves. Their non-ionizing nature, high resolution, deep penetration, and spectral fingerprinting make them promising for biomedical applications [4], [5], [6]. Our main aim was to design a multiband absorber with high absorption value in the THz region for biomedical sensing applications. Multipeak MMA sensors can help work with multiple modes of interest and also reduce error because they generate more sensing data, thus improving the reliability of the data [7]. This octagonal structure is ideal for achieving our goal by providing three distinct peaks in the region 1-6.5 THz with excellent absorption values.

**Reviewer, Concern #7:** The conclusion is in a good form.

**Author response:** Thank you very much, sir, for your valuable comments.

**Reviewer, Concern #8:** Please mention the experimental application possibilities and future directions.

**Author response:** Thank you very much for your comments on our article. Based on your suggestion, we provided the experiment application and future directions before the ***conclusion***.

**Author action:** We updated the manuscript based on reviewer comments.

Although the current study is fully simulation-based and utilises ADS to validate the design using comparable circuit modelling, the suggested structure was created with the feasibility of practical production in mind. The absorber can be fabricated using well-known microfabrication methods, such as inkjet printing or sputtering, which are frequently employed in the terahertz domain to create metallic resonators on both rigid and flexible substrates, as discussed earlier. After the biosensor is manufactured, its sensitivity and absorption performance can be evaluated experimentally with high-precision terahertz time-domain spectroscopy (THz-TDS) [8]. Before actual sample testing, a dry phantom setup that mimics the electromagnetic behaviour of living tissues will be utilised as a preliminary step to fine-tune sensor performance. Subsequent research will focus on utilising the sensor to detect diseases. Human sample testing will require ethical approval, and subsequent clinical trials will include statistical evaluation of diagnostic performance using sensitivity, specificity, and predictive accuracy.

# Reviewer #2:

**Reviewer, Concern #1:** The entire study is simulation-based, using CST Studio Suite and ADS, without experimental fabrication or measurement.

**Author response:** Thank you very much for your comments on our article. We clarify it before ***Conclusion***.

**Author action:** We updated the manuscript based on reviewer comments.

Although the current study is fully simulation-based and utilises ADS to validate the design using comparable circuit modelling, the suggested structure was created with the feasibility of practical production in mind. The absorber can be fabricated using well-known microfabrication methods, such as inkjet printing or sputtering, which are frequently employed in the terahertz domain to create metallic resonators on both rigid and flexible substrates, as discussed earlier.

**Reviewer, Concern #2:** Simulation results, while promising, do not account for real-world fabrication imperfections, material losses, or environmental noise.

**Author response:** Thank you very much for your comments on our article. Based on your suggestion, we have made revisions to the last paragraph of the ***Materials and Methods*** section.

**Author action:** We updated the manuscript based on reviewer comments.

Because this is a thin-film structure, fabricating the absorber presents several challenges. Strict tolerances must be maintained in order to achieve accurate control over micro- and nanoscale dimensions. It may also be challenging to coordinate multiple layers within a complex design accurately. Material losses can also be caused by issues such as dielectric loss during processing, complex mask creation, and material incompatibility. The manufacturing outcome may also be influenced by environmental conditions, such as humidity and temperature [9], [10], [11], [12]. Therefore, to effectively implement this concept in practice, sophisticated fabrication processes are needed.

**Reviewer, Concern #3:** The study focuses on refractive index sensing of a few biological targets (glucose, malaria, HeLa cells). No analysis is provided on how the sensor performs with mixed samples.

**Author response:** Thank you very much for your comments on our article. Based on your suggestions, we have updated after the cancer detection section in our manuscript for better clarity.

**Author action:** We updated the manuscript based on reviewer comments.

Different refractive indices at predetermined intervals, multiple fixed values for individual samples, and a mixed sample of glucose and water were used to assess the sensor's performance. The results of these studies demonstrate that the sensor performs effectively with both mixed and pure biological materials. Furthermore, it has the potential to be applied across various biochemical contexts, thereby advancing technology while enhancing cost-effectiveness and reducing time consumption in research and diagnostics.

**Reviewer, Concern #4:** As we know, the given absorber is a periodic structure. How to put the test object in?

**Author response:** Thank you very much for your comments on our article. Based on your suggestion, we have made revisions to the first paragraph of the ***Sensing performance analysis*** section to provide a clearer explanation.

**Author action:** We updated the manuscript based on reviewer comments.

Fig 10(c) shows the sensor configuration, which includes a 3 µm sensor that can be used for sensing applications. The sensor layer is placed above the substrate, and the top gold resonator layer lies within this configuration, initially left vacant to allow for sample insertion. To introduce the test object, a liquid form of the analyte is dropped or injected into the sensor layer from the top side, allowing it to fill the sensing region. This setup ensures direct interaction between the test sample and the resonator, enabling changes in the dielectric environment to be detected through measurable shifts in the resonance frequency. The experimental setup for this sensor is shown in Fig 10(a).

**Reviewer, Concern #5:** The device operates in 1–6.5 THz, but comparison with existing literature (Table 3, Table 4) often includes devices operating in lower or broader ranges.

**Author response:** Thank you very much for your comments on our article. Based on your suggestion, we have update those table by adding several existing literature work.

**Author action:** We updated the manuscript based on reviewer comments.

Table 3: Comparison of absorption with previous research

| **Ref.** | **Resonance Frequency (THz)** | **Structure Size (µm)** | **Absorption (%)** | **Year** |
|---|---|---|---|---|
| [13] | 3.5-4.1, 7.15 | 35 × 35 | 90, 98.9 | 2019 |
| [14] | 4.59, 6.18 | 2.4 × 2.4 | 99.91, 99.31 | 2019 |
| [15] | 1-3 | - | 97, 98, 99 | 2021 |
| [16] | 2-6 | 4.75 × 4.75 | 98.75 | 2021 |
| [17] | 0-3 | 80 × 80 | 99, 80, 95 | 2022 |
| [18] | 0.66–1.84 | 114 × 114 | 94.4 | 2023 |
| [19] | 2.638, 5.158 | 30 × 30 | Almost 90 | 2024 |
| Proposed | 1.85, 3.62, 5.63 | 41 × 41 | 99.6, 99.6, 99.4 | 2025 |

Table 4: Comparison of Sensitivity performance with recent research

| Ref. | Operating Frequency (THz) | S (THz/RIU) | Year Published | Publication in | Applications |
|---|---|---|---|---|---|
| [20] | 1.4-2.8 | 0.13, 0.14 | 2024 | Photon. Nanostruct. Fundam. Appl. | sensing |
| [21] | 0-4 | 0.103, 0.095 | 2023 | Scientific Reports | Microorganism detection |
| [22] | 1-1.8 | 0.278 | 2023 | Photonics | Bovin serum, albumin protein |

| | | | | | |
|---|---|---|---|---|---|
| [23] | 0-1.2 | 0.0515, 0.076 | 2023 | IEEE Access | Non-melanoma skin cancer detection |
| [24] | 1-1.6 | 0.281 | 2023 | Photonics | biosensor |
| [25] | - | 0.1057 | 2022 | IEEE Sensors | Biomedical sensing |
| [26] | 0-1 | 0.0968, 0.1182 | 2022 | American Chemical Society | Biomedical Application |
| [27] | 0.5-2.5 | 1.21 | 2022 | Nanomaterials | Breast cancer detection |
| [28] | 0.1-3 | 0.3, 0.912 | 2022 | Biosensors | sensor |
| [16] | 2-6 | 0.851 | 2021 | Plasmonics | RI biosensor |
| This Work | 1-6 | 1.5, 1, 0.66 | 2025 | - | Biomedical Application |

**Reviewer, Concern #6:** While some angle-dependent studies are shown, the absorption shifts are noticeable beyond 30°, especially at 60°, where distortions appear.

**Author response:** Thank you very much for your comments on our article. Based on your suggestion, we have made revisions to the first paragraph of the ***Angle stability*** section.

**Author action:** We updated the manuscript based on reviewer comments.

All of the peaks, however, remain until $30^0$ , after which the first and second peaks begin to distort, but we still detected a number of peaks with significant absorption values. These angle shifts are caused by impedance mismatching conditions [29], which result from variations in the interaction position of incident light within the structure. These conditions cause polarisation mismatch and variations in optical path length, particularly at oblique angles like 60°, where these distortions become more noticeable. However, for this kind of incidence angle, we can utilise these peaks if needed, as we also observe many absorption peaks here.

**Reviewer, Concern #7:** How can we improve the performance of the given sensor.

**Author response:** Thank you very much for your comments on our article. Based on your suggestions we update our manuscript before Table 4.

**Author action:** We updated the manuscript based on reviewer comments.

The sensitivity performance of this sensor is compared with that of earlier studies in Table 4. There are various methods to boost this sensor's performance, which can be used to expand its possibilities. Better sensitivity could be achieved by altering the structure or by employing different materials and substrates within the existing arrangement. Its performance can also be improved by modifying the analyte layer's thickness or design parameters.

**Reviewer, Concern #8:** In Fig.1, the basic of information is not clear. The physical model of materials is not given.

**Author response:** Thank you very much for your comments on our article. Based on your suggestion, we have made revisions on the first paragraph of ***Materials and Methods*** section.

**Author action:** We updated the manuscript based on reviewer comments.

Here, gold is used on the front and back sides, which have electrical and thermal conductivities of $4.561 \times 10^7$ S/m and 314 W/K/m, respectively. Gold possesses several physical properties, including a density of 19,320 kg/m³, a specific heat capacity (Cs) of 0.13 J/K/Kg, a Young's modulus of 78 GPa, and a Poisson's ratio of 0.42 [30]. The substrate PTFE is used between these gold layer which has a electrical conductivity of 2.1. PTFE has outstanding thermal resistance and notable flexibility, and its low dielectric permittivity and loss tangent make it well-suited for THz sensing applications [31].

For the x and y axes, the unit cell boundary condition is applied, and an open boundary condition is applied to the z-axis, the structure with directions shown in Fig 1. In the upper-left part of this figure, the polarization angle (ϕ) and the incident angle (θ) of the incoming wave are both set to 0 degrees, meaning the plane wave is normally incident along the z-axis with the electric field (E) polarized along the x-axis and the magnetic field (H) oriented along the y-axis.

**Reviewer, Concern #9:** Please highlight the advance of the study in Introduction. Please explain the development and creative work. The literature review should be carefully considered.

**Author response:** Thank you very much for your comments on our article. Based on your suggestion, we have update the last paragraph of the ***Introduction*** section.

**Author action:** We updated the manuscript based on reviewer comments.

In these studies, many researchers have proposed various metamaterial-based structures and sensors for biomedical applications. However, most of these designs face certain limitations. Some operate only in a single frequency band, while others demonstrate multiple absorption peaks but utilize only one for sensing. In many cases, placing a sensing layer alters the resonance characteristics or causes unstable performance when the refractive index changes. In contrast, a multi-band metamaterial absorber (MMA) enhances sensing reliability and reduces measurement error [7] by providing a consistent response across multiple distinct resonance modes. To overcome these issues, our study presents a novel, triple-band metamaterial absorber (MMA) with a compact footprint of $41 \times 41\,\mu m^2$, which is designed without altering the commonly used substrate and materials. This development demonstrates creative structural innovation through precise geometrical tuning and multilayered symmetry that enables high absorption rates (above 99%) at all three resonant frequencies. A comprehensive parametric study was conducted to optimize the structure for maximum performance. Furthermore, E-field, H-field, and surface current distributions were analysed. A sensing layer was introduced to evaluate the sensor's refractive index sensitivity across all three peaks, which remained stable and showed high sensitivity. We further demonstrated the sensor's practical utility by detecting biological samples, including glucose, malaria-infected cells, and cervical cancer cells. Moreover, the sensor's suitability for microwave imaging (MWI) applications was also analysed. Overall, the sensor's high absorption, structural stability, and intense sensitivity make it a promising candidate for biomedical applications.

**Reviewer, Concern #10:** Please polish the abstract. Please check the logic of abstract. Please add sentences to explain the meaning, the main points, the improvement and the promising application of the study. Plenty of detail data have given, however, in abstract, important

procedures and results should be mentioned in simple manner. Please focus on the main points and the improvement of the study.

**Author response:** Thank you very much for your comments on our article. Based on your suggestion, we have carefully polished the abstract for clarity and logical flow. We added sentences to better explain the key points, including the main objectives, improvements over previous work, and the promising applications of our study. Specifically, we emphasized the absorber's high absorption rates and multi-band sensitivity as the central contributions. Additionally, we briefly mentioned the procedures (simulation, circuit modeling, and field analysis) and presented major results (absorption values, sensitivity, Q-factor, and FoM) in a concise and accessible manner. The suitability for both biochemical sensing and microwave imaging applications was also included to reflect the scope and impact of the proposed work.

**Author action:** We updated the manuscript based on reviewer comments.

Due to the growing interest in metamaterials for biomedical applications, this study presents the design and analysis of a novel, compact, triple-band metamaterial absorber for biological sensing in the terahertz range. The structure, with dimensions of $41 \times 41\ \mu m^2$, exhibits exceptionally high absorption rates above 99% at three distinct resonance frequencies of 1.85, 3.62, and 5.63 THz. To validate the design, an equivalent circuit model was created and evaluated alongside electric field, magnetic field, and surface current distributions. A key focus of this work is the sensor's performance, evaluated by introducing a sensing layer with varying refractive indices. The absorber demonstrates outstanding sensitivity values of 1.5 THz/RIU, 1 THz/RIU, and 0.66 THz/RIU at the third, second, and first resonances, respectively, indicating highly reliable sensing performance across multiple frequency bands. Furthermore, its suitability for biochemical applications was evaluated by testing its ability to detect different samples, such as glucose, malaria, and cervical cancer cells. The absorber also demonstrates strong performance metrics, with a maximum quality factor of 39.13 and a figure of merit (FoM) of 6.95, supporting its reliability. Additionally, its suitability for microwave imaging (MWI) technology is also examined. The combination of near-perfect absorption and high sensitivity makes this compact metamaterial absorber a promising candidate for advanced biomedical sensing and diagnostic technologies.

**Reviewer, Concern #11:** English in this paper needs improvement, which can make this paper more like a journal paper.

**Author response:** Thank you very much for your comments on our article. Based on your suggestions we improve the English of this manuscript.

**Reviewer, Concern #12:** Many information without references.

**Author response:** Thank you very much for your comments on our article. Based on your suggestions, we have carefully reviewed the manuscript and added references in all relevant sections where needed. We appreciate your guidance, which has helped improve the clarity and credibility of our work.

**Reviewer, Concern #13:** The author should elaborate all results with reason.

**Author response:** Thank you very much for your comments on our article. Based on your suggestions we update in first and last paragraph of ***Absorption analysis*** section, fourth paragraph of the ***Sensing performance analysis*** section, and before Table 5.

**Author action:** We updated the manuscript based on reviewer comments.

Absorption is a crucial parameter, as high absorption in a metamaterial absorber ensures maximum interaction with the incident electromagnetic wave.

Furthermore, we calculate the Q-factor value for these peaks because the quality factor is an important parameter for evaluating frequency mode performance. It also helps to identify how this device is effective in sensing applications [32]. This, in turn, helps to evaluate the overall effectiveness of the device in sensing performance.

Sensitivity is a key parameter that defines the sensor's ability to detect and differentiate minor variations in a sample's permittivity [33]. Higher sensitivity values at each resonance demonstrate the device's strong interaction with the analyte, enabling reliable detection performance.

The figure of merit (FOM) is a crucial element for comparing the sensing capabilities of different sensors [9].Our relatively high FOM values confirm that the proposed absorber performs competitively with or better than many existing THz sensors.

**Reviewer, Concern #14:** For the explanation of sensors, please add the extra contents or theory to analyze.

**Author response:** Thank you very much for your comments on our article. Based on your suggestions we added the following text in the second paragraph of ***Sensing performance analysis*** section.

**Author action:** We updated the manuscript based on reviewer comments.

We must examine certain crucial electromagnetic characteristics, such as effective permittivity ($\varepsilon_{eff}$), effective permeability ($\mu_{eff}$), refractive index (n), and wave impedance (z), to understand the metamaterial's behaviour. These variables are essential to understanding the sensor layer. They can be computed using several well-established methods, including the Lossy-Drude models, the Nicolson-Ross-Weir method, and the transmission-reflection (TR) method. The following formula is used to determine n in both the Nicolson-Ross-Weir and Lossy-Drude methods: $n = \cos^{-1}(1/k\delta)((1 - S_{11} + S_{22}) / (2S_{21}))$. The wave impedance z is obtained as follows: $z = \text{sqrt}[((1 + S_{11})^2 - S_{22})] / [(1 - S_{11})^2 - S_{22})$. Particularly when multiple sensing layers are present, these derived characteristics help describe the electromagnetic response of the structure [34].

1. https://www.nature.com/articles/s41598-023-46363-x

**Reviewer, Concern #15:** It is recommended to cite the following articles:

a. https://www.nature.com/articles/s41598-023-46363-x
b. https://iopscience.iop.org/article/10.1088/2040-8986/ac8889/meta
c. https://www.sciencedirect.com/science/article/pii/S2211379720320106
d. https://link.springer.com/chapter/10.1007/978-981-97-0142-1_35

**Author response:** Thank you very much for your comments on our article. Based on your suggestions we have added the suggested literature in the in the introduction section and in the reference section.

**Author action:** We updated the manuscript based on reviewer comments.

For use in microwave sensing applications, an Archimedes spiral-shaped metamaterial (MTM) absorber was developed [34]. With a high quality factor of 84.5 and a maximum absorption of 99.9%, the absorber displays seven resonance peaks. The proposal is for a triple-band mtm structure for terahertz applications [35]. For the three peak frequencies, this arrangement exhibits remarkable absorption values of 99%, 99%, and 100%, with peak sensitivities of 2.76 THz $\mu m^{-1}$ for analyte thickness and 1.55 THz $RIU^{-1}$ for refractive index detection. For RI index sensing applications, an ultra-narrow perfect mtm absorber in the thz region was proposed [36]. The study's greatest q-factor and absorption were 637 and 99.49%, respectively, and its sensitivity value was 506 $RIU^{-1}$. For the aim of sensing cooking oils, a metamaterial structure based on S.S.R.R. is shown [37]. At 3.04 and 3.44 GHz, the absorption values are 99.9 and 99.7, respectively, and the sensitivity values are 1410.29 and 1148.16.

**References:**


[1] Y. I. Abdulkarim *et al.*, "A thermally stable and polarization insensitive square-shaped water metamaterial with ultra-broadband absorption," *J. Mater. Res. Technol.*, vol. 13, pp. 1150–1158, 2021.

[2] Y. I. Abdulkarim *et al.*, "Simulation and lithographic fabrication of a triple band terahertz metamaterial absorber coated on flexible polyethylene terephthalate substrate," *Opt. Mater. Express*, vol. 12, no. 1, pp. 338–359, 2021.

[3] M. Erdogan Alkurt, E. Unal, M. Karaaslan, O. Akgol, and M. Aksoy, "Ultrathin photonic absorber with wideband polarization independency characteristics using fractal combinations of ring resonators on ZnSe substrate," *Opt. Eng.*, vol. 63, no. 11, pp. 115102–115102, 2024.

[4] E. Pickwell and V. P. Wallace, "Biomedical applications of terahertz technology," *J. Phys. Appl. Phys.*, vol. 39, no. 17, p. R301, 2006.

[5] M. Danciu *et al.*, "Terahertz spectroscopy and imaging: a cutting-edge method for diagnosing digestive cancers," *Materials*, vol. 12, no. 9, p. 1519, 2019.

[6] Y. Peng, C. Shi, Y. Zhu, M. Gu, and S. Zhuang, "Terahertz spectroscopy in biomedical field: a review on signal-to-noise ratio improvement," *PhotoniX*, vol. 1, pp. 1–18, 2020.

[7] R. Jian, S. Wu, B. Zhao, and G. Xiong, "Tunable multi-peak perfect absorbers based on borophene for high-performance near-infrared refractive index sensing," *Opt. Mater.*, vol. 131, p. 112751, 2022.

[8] S. J. Park *et al.*, "Detection of microorganisms using terahertz metamaterials," *Sci. Rep.*, vol. 4, no. 1, p. 4988, 2014.

[9] S. J. Mishu, M. A. Rahman, and N. Dhar, "Highly sensitive refractive index sensing with a dual-band optically transparent ITO-based perfect metamaterial absorber for biomedical applications," *Heliyon*, vol. 10, no. 5, 2024.

[10] J. S. Melinger, S. S. Harsha, N. Laman, and D. Grischkowsky, "Temperature dependent characterization of terahertz vibrations of explosives and related threat materials," *Opt. Express*, vol. 18, no. 26, pp. 27238–27250, 2010.

[11] M. Decker *et al.*, "High-efficiency dielectric Huygens' surfaces," *Adv. Opt. Mater.*, vol. 3, no. 6, pp. 813–820, 2015.

[12] M. Cingolani, G. Fusaro, and M. Garai, "The influence of thermo-hygrometric conditions on metamaterials' acoustic performance: an investigation on a 3D printed coiled-up resonator," in *INTER-NOISE and NOISE-CON Congress and Conference Proceedings*, Institute of Noise Control Engineering, 2023, pp. 3045–3050.

[13] Y. Zhang *et al.*, "Dual-band switchable terahertz metamaterial absorber based on metal nanostructure," *Results Phys.*, vol. 14, p. 102422, 2019.

[14] H. Li, J. Niu, and G. Wang, "Dual-band, polarization-insensitive metamaterial perfect absorber based on monolayer graphene in the mid-infrared range," *Results Phys.*, vol. 13, p. 102313, 2019.

[15] Y. Wang *et al.*, "Terahertz tunable three band narrowband perfect absorber based on Dirac semimetal," *Phys. E Low-Dimens. Syst. Nanostructures*, vol. 131, p. 114750, 2021.
[16] M.-R. Nickpay, M. Danaie, and A. Shahzadi, "Highly sensitive THz refractive index sensor based on folded split-ring metamaterial graphene resonators," *Plasmonics*, pp. 1–12, 2021.
[17] S. Zhuang *et al.*, "Graphene-based absorption–transmission multi-functional tunable THz metamaterials," *Micromachines*, vol. 13, no. 8, p. 1239, 2022.
[18] L. Gevorgyan, H. Haroyan, H. Parsamyan, and K. Nerkararyan, "Tunable ultra-broadband terahertz metamaterial absorber based on vanadium dioxide strips," *RSC Adv.*, vol. 13, no. 18, pp. 11948–11958, 2023.
[19] N. Anjum and A. K. Paul, "Design and Optimization of a Metamaterial Absorber for Solar Energy Harvesting in the THz Frequency Range," *ArXiv Prepr. ArXiv241015654*, 2024.
[20] N. Singh, R. Dhara, and S. Yadav, "Design and optimization of a polarization-insensitive terahertz metamaterial absorber for sensing applications," *Photonics Nanostructures-Fundam. Appl.*, vol. 62, p. 101314, 2024.
[21] R. Bhati and A. K. Malik, "Multiband terahertz metamaterial perfect absorber for microorganisms detection," *Sci. Rep.*, vol. 13, no. 1, p. 19685, 2023.
[22] Y. Shen *et al.*, "Low-concentration biological sample detection using an asymmetric split resonator terahertz metamaterial," in *Photonics*, MDPI, 2023, p. 111.
[23] M. N. Hamza and M. T. Islam, "Designing an extremely tiny dual-band biosensor based on MTMs in the terahertz region as a perfect absorber for non-melanoma skin cancer diagnostics," *IEEE Access*, vol. 11, pp. 136770–136781, 2023.
[24] M. Guan, X. Sun, J. Wei, X. Jia, X. Cheng, and R. Cheng, "High-sensitivity terahertz biosensor based on plasmon-induced transparency metamaterials," in *Photonics*, MDPI, 2023, p. 1258.
[25] H. Singh, A. Gupta, R. S. Kaler, S. Singh, and A. S. Gill, "Designing and analysis of ultrathin metamaterial absorber for W band biomedical sensing application," *IEEE Sens. J.*, vol. 22, no. 11, pp. 10524–10531, 2022.
[26] Y. I. Abdulkarim *et al.*, "Highly sensitive dual-band terahertz metamaterial absorber for biomedical applications: simulation and experiment," *ACS Omega*, vol. 7, no. 42, pp. 38094–38104, 2022.
[27] C. Tan *et al.*, "Cancer diagnosis using terahertz-graphene-metasurface-based biosensor with dual-resonance response," *Nanomaterials*, vol. 12, no. 21, p. 3889, 2022.
[28] X. Lu, H. Ge, Y. Jiang, and Y. Zhang, "A dual-band high-sensitivity THz metamaterial sensor based on split metal stacking ring," *Biosensors*, vol. 12, no. 7, p. 471, 2022.
[29] Q. Zhou *et al.*, "Metasurface terahertz perfect absorber with strong multi-frequency selectivity," *ACS Omega*, vol. 7, no. 41, pp. 36712–36727, 2022.
[30] S. A. Haque, M. Ahmed, A. Alqahtani, M. R. Maruf, M. T. Islam, and M. Samsuzzaman, "Modelling and analysis of a triple-band metamaterial absorber for early-stage cervical cancer HeLa cell detection," *Opt. Lasers Eng.*, vol. 181, p. 108426, 2024.
[31] S. Asgari and T. Fabritius, "Multi-band terahertz metamaterial absorber composed of concentric square patch and ring resonator array," *Opt. Contin.*, vol. 3, no. 2, pp. 148–163, 2024.
[32] P. Jain *et al.*, "Machine learning assisted hepta band THz metamaterial absorber for biomedical applications," *Sci. Rep.*, vol. 13, no. 1, p. 1792, 2023.
[33] Q. Jiang, Y. Yu, Y. Zhao, Y. Zhang, L. Liu, and Z. Li, "Ultra-compact effective localized surface plasmonic sensor for permittivity measurement of aqueous ethanol solution with high sensitivity," *IEEE Trans. Instrum. Meas.*, vol. 70, pp. 1–9, 2021.
[34] O. S. Lateef, M. Al-Badri, K. S. L. Al-Badri, and S. A. Mohammed, "Polarization-insensitive Archimedes'-spiral-shaped ultrathin metamaterial absorbers for microwave sensing application," *Sci. Rep.*, vol. 13, no. 1, p. 19445, 2023.
[35] Y. I. Abdulkarim *et al.*, "A polarization-insensitive triple-band perfect metamaterial absorber incorporating ZnSe for terahertz sensing," *J. Opt.*, vol. 24, no. 10, p. 105102, 2022.
[36] D. Hu, T. Meng, H. Wang, Y. Ma, and Q. Zhu, "Ultra-narrow-band terahertz perfect metamaterial absorber for refractive index sensing application," *Results Phys.*, vol. 19, p. 103567, 2020.
[37] M. A. Khalil, W. H. Yong, M. T. Islam, A. Hoque, M. R. Islam, and M. S. Islam, "A perfect metamaterial absorber for sensing application of edible oil," in *International Conference on Space Science and Communication*, Springer, 2023, pp. 357–364.
